# Atorvastatin Promotes Pro/anti-inflammatory Phenotypic Transformation of Microglia via Wnt/β-catenin Pathway in Hypoxic-Ischemic Neonatal Rats

**DOI:** 10.1007/s12035-023-03777-y

**Published:** 2023-11-24

**Authors:** Luting Yu, Lingyi Huang, Yuanyuan Zhao, Shixi Liu, Ruixi Zhou, Yan Yue, Hao Sun, Xiaojuan Su, Qian Liu, Shiping Li, Junjie Ying, Fengyan Zhao, Yi Qu

**Affiliations:** 1 Department of Pediatrics, Key Laboratory of Birth Defects and Related Diseases of Women and Children (Ministry of Education), NHC Key Laboratory of Chronobiology, West China Second University Hospital, Sichuan University, Chengdu, 610041 Sichuan China; 2 Department of Orthodontics, State Key Laboratory of Oral Diseases, West China College of Stomatology, Sichuan University, Chengdu, 610041 China

**Keywords:** Hypoxic ischemic, Microglia, Pro/anti-inflammatory phenotype, Sclerostin, Wnt/β-catenin pathway

## Abstract

Inflammatory reaction plays a key role in the pathogenesis of hypoxic-ischemic encephalopathy (HIE) in neonates. Microglia are resident innate immune cells in the central nervous system and are profoundly involved in neuroinflammation. Studies have revealed that atorvastatin exerts a neuroprotective effect by regulating neuroinflammation in adult animal models of brain stroke and traumatic brain injury, but its role regarding damage to the developing brain remains unclear. This study aimed to clarify the effect and mechanism of atorvastatin on the regulation of microglia function in neonatal hypoxic-ischemic brain damage (HIBD). The oxygen glucose deprivation (OGD) of microglia and neonatal rat HIBD model was established. Atorvastatin, recombinant sclerostin protein (SOST), and XAV939 (degradation of β-catenin) were administered to OGD microglia and HIBD rats. The pathological changes of brain tissue, cerebral infarction volume, learning and memory ability of rats, pro-inflammatory (CD16^+^/Iba1^+^) and anti-inflammatory (CD206^+^/Iba1^+^) microglia markers, inflammation-related indicators (Inos, Tnfα, Il6, Arg1, Tgfb, and Mrc1), and Wnt/β-catenin signaling molecules were examined. Atorvastatin reduced OGD-induced pro-inflammatory microglia and pro-inflammatory factors, while increasing anti-inflammatory microglia and anti-inflammatory factors. In vivo, atorvastatin attenuated hypoxia-ischemia (HI)-induced neuroinflammation and brain damage. Mechanistically, atorvastatin decreased SOST expression and activated the Wnt/β-catenin signaling pathway, and the administration of recombinant SOST protein or XAV939 inhibited Wnt/β-catenin signaling and attenuated the anti-inflammatory effect of atorvastatin. Atorvastatin promotes the pro/anti-inflammatory phenotypic transformation of microglia via the Wnt/β-catenin pathway in HI neonatal rats. Atorvastatin may be developed as a potent agent for the treatment of HIE in neonates.

## Introduction

Neonatal hypoxic-ischemic encephalopathy (HIE) refers to hypoxic-ischemic brain damage (HIBD) caused by hypoxic asphyxia during the perinatal period, causing a series of characteristic neuropathological changes and clinical manifestations [1–3]. In middle-low income countries, the incidence of neonatal HIE is approximately 9 per 1000 live births [4]. In developed countries, the incidence of neonatal HIE is 2–3 per 1000 live births [5]. Surviving children have serious sequelae, such as cerebral palsy, cognitive dysfunction, epilepsy, and delayed brain development [6–8].

Microglia are resident innate immune cells in the central nervous system (CNS), which play a fundamental role in monitoring and responding to invading pathogens and environmental damage [9]. Microglia are widely distributed in the CNS, accounting for approximately 10% of the total cells in the CNS [10–12]. Microglia can be divided into pro-inflammatory and anti-inflammatory phenotypes [13] and can undergo functional transformation between pro-inflammatory and anti-inflammatory [14]. Pro-inflammatory microglia mainly express surface antigens such as CD16 and CD86 [15] and produce a large number of cytotoxic substances such as interleukin (IL) 6, tumor necrosis factor α (TNFα), and the inducible nitric oxide synthase (iNOS), thereby exerting toxic effects on neurons and other glial cells [16]. Anti-inflammatory microglia primarily express surface antigens such as arginase 1 (Arg1) and mannose receptor C-type 1 (Mrc1, also known as CD206) [17] and release anti-inflammatory factors including IL4 and transforming growth factor β (TGFβ), thereby performing a neuroprotective role [18]. Microglia-mediated neuroinflammation has been implicated in various diseases such as Alzheimer’s disease, Parkinson’s disease, multiple sclerosis, and cerebral ischemic injury [19–21].

Increasing evidences indicate that hypoxic-ischemic (HI)-induced neuroinflammation is the main factor leading to the pathological changes of neonatal brain injury [22–24]. Elevated pro-inflammatory cytokines such as IL6, IL8, and IL1β were identified in the cerebrospinal fluid of infants with HIE, which were associated with poor neurological outcomes and the development of cerebral palsy [23–27]. As inherent innate immune cells, microglia activation is a sign of brain neuroinflammation and plays a key role in hypoxic-ischemic brain damage (HIBD) [28–30]. Studies have shown that HI increases pro-inflammatory microglia in the brain, reduces anti-inflammatory microglia, promotes the release of inflammatory factors, and causes brain damage [31]. Therefore, drugs aimed at inhibiting the inflammatory response are expected to be an effective treatment for improving HIBD.

Atorvastatin is a statin that inhibits 3-hydroxy-3-methylglutaryl coenzyme A (HMG-CoA) reductase and can be used to reduce cholesterol and the risk of cardiovascular disease [32]. Studies have revealed that atorvastatin also played a neuroprotective role by reducing inflammation. Atorvastatin reduced the microglial activation and leukocyte adhesion and infiltration in adult mouse and rat models of middle cerebral artery occlusion [33, 34]. Besides, in an adult mouse traumatic brain injury model, atorvastatin increased the anti-inflammatory microglia and attenuated brain injury [35]. However, these studies all focused on adult animal brain injury models, and the role and mechanism of atorvastatin in developmental brain injury remains unclear. In this study, we will establish microglial oxygen glucose deprivation (OGD) model and neonatal rat HIBD model and explore the effect of atorvastatin on regulating the function of microglia. It has been suggested that atorvastatin can regulate the function of microglia via activating the Wnt/β-catenin signaling pathway [36–38]. Besides, Matias et al. have found that Wnt/β-catenin signaling can regulate the pro/anti-inflammatory phenotypic transformation of microglia [39, 40]. Therefore, mechanistically, we will focus on assessing whether atorvastatin regulates microglia function in HIBD through Wnt/β-catenin signaling pathway.

## Materials and Methods

### Drugs and Reagents

Atorvastatin (344423) and 2,3,5-triphenyltetrazolium chloride monohydrate (TTC) (T8877) were purchased from Sigma-Aldrich (St. Louis, MO, USA). Atorvastatin was dissolved with DMSO and then diluted with ultrapure water to the desired concentration. The final concentration of DMSO in the atorvastatin solution administered to the animals is 0.5%. The total RNA extraction kit (LS1040) was purchased from Promega Biotechnology Co., Ltd. (Beijing, China). SYBR (Q711) was purchased from Vazyme Biotech Co., Ltd. (Nanjing, China). Recombinant mouse sclerostin (SOST) protein (1589-ST), which can inhibit Wnt/β-catenin signaling pathway by antagonizing the binding of Wnt to its receptor and reducing the expression of downstream β-catenin [41, 42], was purchased from R&D Systems (Minnesota, MN, USA). XAV939 (S1180), which can inhibit Wnt/β-catenin signaling pathway by degrading β-catenin, was purchased from American Selleck Biotechnology Co., Ltd. (China Branch, Shanghai). β-actin (20068) was purchased from Zen Bioscience Co., Ltd. (Chengdu, China). H3 (ab1791) and β-catenin (ab32572) were purchased from Abcam (Cambridge, UK). GSK3β (12456) and p-GSK3β (9322) were purchased from CST (Boston, USA).

### Cell Culture, Treatment, and Viability Assay

The cerebral cortices of 1-day-old rats were digested using papain (2 mg/ml) for 30 min; the cell filtrate was collected using a 70-μm filter and inoculated with culture medium (89% DMEM, 10% FBS, and 1% penicillin/streptomycin). Each T75 culture flask was inoculated with 2 × 10^7^ cells. When the mixed cells were overgrown, the culture flask was placed on a shaker, and the temperature was set to 37 °C; the flask was shaken at 180 rpm for 40 min, collected in the culture flask, and centrifuged (1000 rpm, 10 min). The supernatant was discarded, and the pellet was microglia. Each 6-well plate was inoculated with 1 × 10^6^ cells. The control group was cultured in a normal incubator (37 °C, 5% CO_2_). The OGD model was established using DMEM sugar-free medium (1% O_2_, 5% CO_2_, and 94% N_2_) in a three-gas incubator for 8 h. The cells were treated with atorvastatin (1 μM), recombinant SOST protein (50 ng/ml), and XAV939 (1 μM) for 24 h before subsequent experiments.

Atorvastatin with 0.1, 1, and 10 μM was used for cell viability assays. Cells were seeded into 96-well plates, and 10 μl CCK8 solution was added to each well. The cells were cultured in an incubator for 90 min, and the activity of the cells was calculated by measuring the absorbance at 450 nm using a microplate reader.

### Transcriptome Sequencing of Microglia

Total RNA was extracted using a TRIzol RNA extraction kit (Invitrogen, USA) from the cultured microglia. RNA purity and quantification were evaluated using the NanoDrop 2000 spectrophotometer (Thermo Scientific, USA). RNA integrity was assessed using the Agilent 2100 Bioanalyzer. Then, the libraries were constructed using VAHTS Universal V6 RNA-seq Library Prep Kit according to the manufacturer’s instructions. The libraries were sequenced on an llumina NovaSeq 6000 platform, and 150 bp paired-end reads were generated. Differential expression analysis was performed using the DESeq2. *Q* value < 0.05 and fold change > 2 or fold change < 0.5 were set as the threshold for significantly differential expression gene (DEGs).

### HIBD Model and Treatment

Animals were kept in the same room with an environmental temperature of 18–22 °C, a relative humidity of 40–60%, a 12-h/12-h light–darkness cycle, and free access to food and water. All the animal experiments involved in this study have been approved by the Animal Ethics Review Committee of Sichuan University (Animal Ethics Review Pass No.WCSUH21-2018-034). Pregnant rats were purchased from Chengdu Dashuo Experimental Animals. We divided the 7-day-old Sprague Dawley (SD) rats into three groups: sham, HI, and HI+atorvastatin groups. We anesthetized animals with isoflurane with the dosage of 2%. The HIBD model was induced by ligation of the right common carotid artery and then hypoxia (8% O_2_, 92% N_2_) in a hypoxia chamber for 2 h. We first weighed the rats and calculated the dosage required by the rats according to the standard of 10 mg/kg. The corresponding amount is then drawn with a syringe and pumped through a gastric tube. The HI+atorvastatin group was intragastrically administered with atorvastatin (10 mg/kg/d), and the sham and HI groups were administered the corresponding solvents. After 72 h, the rats were anesthetized. Brain tissues were subjected to H&E, TTC, and immunofluorescence staining. The cerebral cortex was used to extract the proteins and RNA for further detection.

### H&E Staining

The brain tissue was fixed with 4% paraformaldehyde, dehydrated with alcohol, soaked in xylene, and embedded in paraffin. The samples were cut into 5-μm slices and dried, dewaxed with xylene, treated with alcohol, then stained with hematoxylin-eosin. An optical microscope (Olympus) was used to observe pathological changes in the sections.

### Infarct Volume Measurement

Each brain was cut into four pieces, and the thickness of each slice was 2 mm. The slices were then placed in 2% TTC solution and incubated at 37 °C for 20 min; the TTC solution was removed, fixed with 4% paraformaldehyde, and photographed. Image-Pro Plus software (version 6.0) was used to calculate. Infarct volume/brain volume was used to determine the percentage of infarct.

### Morris Water Maze Test

Rats used in the Morris water maze test were 28-day old. The escape time (escape incubation period) of the rats to the platform was recorded four times a day for five consecutive days. On day 6, the platform was removed, and each rat swam freely in the maze for 90 s. The number of times each rat passed through the previous escape platform area was recorded.

### Immunofluorescence Staining

We fixed the brain with paraformaldehyde, dehydrated it with sucrose, embedded it with OCT, frozen it in liquid nitrogen, and then stored it at −80 °C for subsequent sections. The slices (10 μm) were soaked in PBS for 10 min to remove the OCT, and then Triton X-100 was used to penetrate the membrane. Primary antibody (150 μl, 1:200) was added to each slide and incubated overnight. The sections were then washed with PBS, and secondary antibodies were added. Finally, the sections were stained with DAPI, washed with PBS, and sealed with an anti-quenching agent. A fluorescence microscope was used for observation and photography.

### Total RNA Extract and RT-qPCR Assay

Total RNA was extracted using the total RNA extraction kit instructions. The primers for *Il6*, *Inos*, *Tnfα*, *Arg1*, *Mrc1*, *Tgfb*, and *β-actin* are shown in Table 1 and were synthesized by Tsingke Biotechnology Co., Ltd.

**Table 1:** Oligonucleotide primers and PCR conditions used for real-time quantitative PCR

Gene	Forward primer	Reverse primer	Product (bp)	Annealing (°C)
Rat				
*Il6*	ATTCTGTCTCGAGCCCACCA	CTGAAGGGCAGATGGAGTTGA	51	60
*Inos*	AGATCCCGAAACGCTACACTT	TGCGGCTGGACTTCTCACTC	174	60
*Tnfα*	GCATGATCCGAGATGTGGAACTGG	CGCCACGAGCAGGAATGAGAAG	113	60
*Arg1*	ACATCAACACTCCGCTGACAACC	GCCGATGTACACGATGTCCTTGG	152	60
*Mrc1*	GAGGACTGCGTGGTGATGAA	CATGCCGTTTCCAGCCTTTC	154	60
*Tgfb*	CTGCTGACCCCCACTGATAC	AGCCCTGTATTCCGTCTCCT	94	60
*β-actin*	ACCCGCCACCAGTTCGC	CACGATGGAGGGGAAGACG	123	60

*IL6* interleukin 6, *Inos* inducible nitric oxide synthase, *Tnfα* tumor necrosis factor α, *Arg1* arginase 1, *Mrc1* mannose receptor C-type 1, *Tgfb* transforming growth factor β

### ELISA

The concentrations of inflammatory factors in cell supernatant and cerebral cortex were determined by microplate reader. iNOS ELISA kit (R0520c, Elabsciences), TNFα ELISA kit (R2856c, Elabsciences), ARG1 ELISA kit (R0070c, Elabsciences), and TGFβ ELISA kit (R1015c, Elabsciences) were used according to the manufacturer’s instructions.

### Capillary Wes Analyses

The cerebral cortex and microglia were lysed with RIPA lysis buffer containing protease and phosphatase inhibitors to extract total protein. Cytoplasmic and nuclear proteins were extracted according to the manufacturer’s instructions. Protein concentration was determined using the BCA protein detection kit, and a western system (Wes-Protein Simple) based on capillary electrophoresis technology was used to quantify the levels of p-GSK3β (1:50), GSK3β (1:50), β-catenin (1:50), H3 (1:200), and β-actin (1:500).

### Statistical Analysis

All experimental data were analyzed using the SPSS software (version 24.0; SPSS Inc., Chicago, IL, USA). The statistical data conforms to the normal distribution. Data are presented as the mean ± SEM. Multiple comparisons between groups were performed using analysis of variance, followed by a post-hoc Tukey test. Differences were considered statistically significant at *p* < 0.05, ^*^p < 0.05, ^**^p < 0.01.

## Results

### Effects of Atorvastatin on the Phenotypic Transformation of Pro-inflammatory and Anti-inflammatory Microglia

**Fig. 1:**
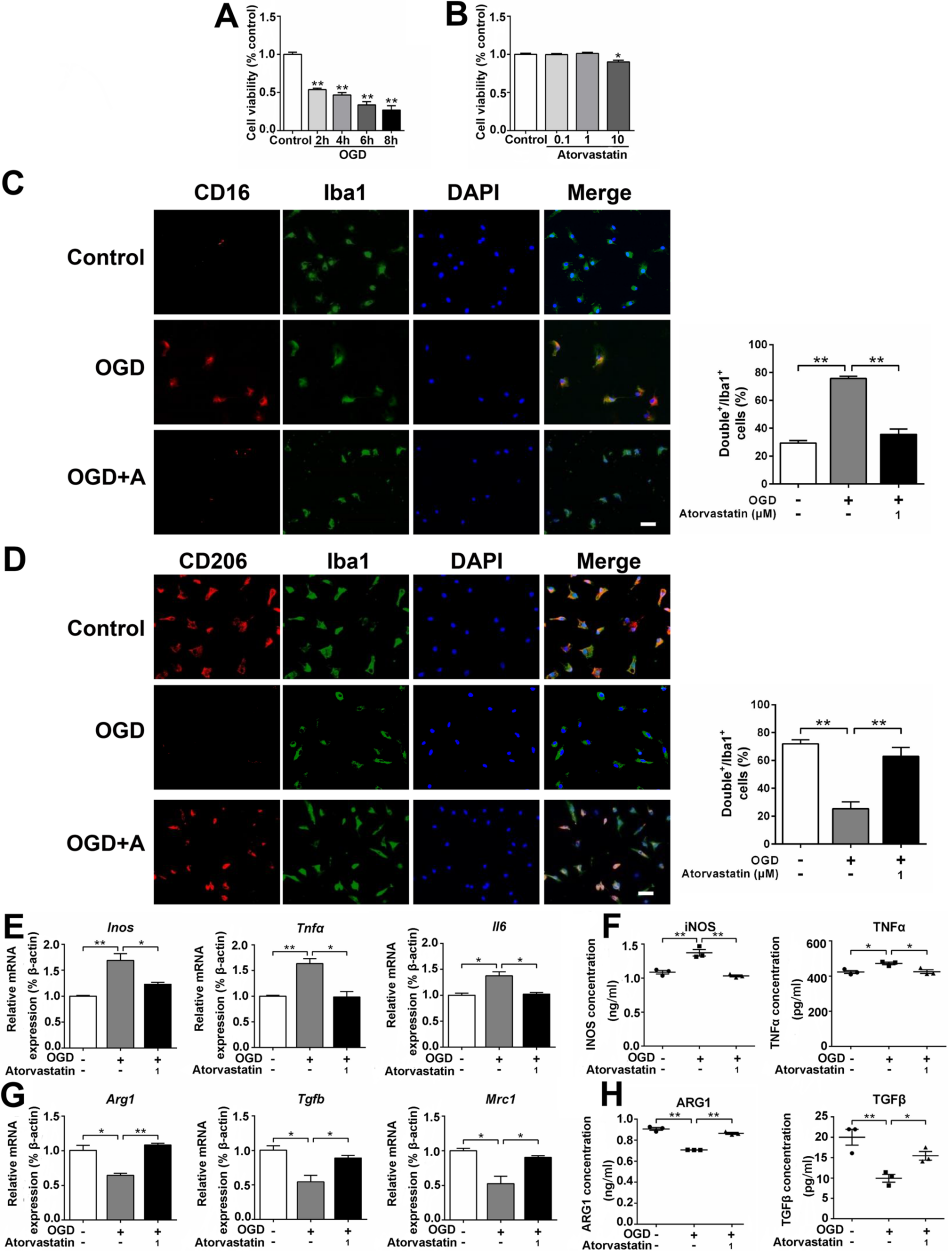
Effects of atorvastatin on the phenotypic transformation of pro-inflammatory and anti-inflammatory microglia. **A**, **B** Cells were treated with different times of OGD (2, 4, 6, and 8 h) and different concentrations of atorvastatin (0.1–10 μM) for 24 h. Cell viability was measured using the CCK-8 assay, *n* = 6/group. **C** Representative photographs of double immunofluorescence staining of CD16 (red) and Iba1 (green). Scale bar = 50 μm, *n* = 6/group. **D** Representative photographs of double immunofluorescence staining of CD206 (red) and Iba1 (green). Scale bar = 50 μm, *n* = 6/group. **E**, **G** The mRNA levels of *Inos*, *Tnfα*, *Il6*, *Arg1*, *Tgfb*, and *Mrc1* were detected by RT-qPCR, *n* = 3/group. **F**, **H** The cell culture supernatant protein levels of iNOS, TNFα, ARG1, and TGFβ were detected by ELISA, *n* = 3/group. Values represent the mean ± SEM. ^*^p < 0.05, ^**^p < 0.01 vs. the respective control

OGD tests for 2, 4, 6, and 8 h showed that 8-h OGD inhibited 73% cell viability; therefore, OGD for 8 h was determined for subsequent experiments (Fig. 1A). Atorvastatin (0.1, 1, and 10 μM) was administered to treat microglia under normal culture conditions, and the results showed that 10 μM atorvastatin affected cell survival, whereas 0.1 and 1 μM atorvastatin had no effect on cell survival, so 1 μM atorvastatin was selected for subsequent experiments (Fig. 1B). Immunofluorescence staining showed that compared with the control group, the pro-inflammatory microglia (CD16^+^/Iba1^+^) increased, while anti-inflammatory microglia (CD206^+^/Iba1^+^) decreased in the OGD group, and atorvastatin administration reduced pro-inflammatory microglia and increased anti-inflammatory microglia (Fig. 1C, D).

RT-qPCR assay showed that compared with the control group, the mRNA levels of pro-inflammatory factors (*Inos*, *Tnfα*, *and Il6*) increased in the OGD group but decreased after atorvastatin administration (Fig. 1E). The mRNA levels of anti-inflammatory factors (*Arg1*, *Tgfb*, *and Mrc1*) decreased in the OGD group compared with the control group but increased after atorvastatin administration (Fig. 1G). ELISA detection of cell culture supernatant showed that compared with the control group, the protein levels of pro-inflammatory factors (iNOS and TNFα) increased in the OGD group but decreased after atorvastatin administration (Fig. 1F). The protein levels of anti-inflammatory factors (ARG1 and TGFβ) decreased in the OGD group compared with the control group but increased after atorvastatin administration (Fig. 1H). It showed that atorvastatin reduced OGD-induced pro-inflammatory microglia and pro-inflammatory factors, while increasing anti-inflammatory microglia and anti-inflammatory factors.

### Sequencing Analysis of Microglia Transcriptome

**Fig. 2:**
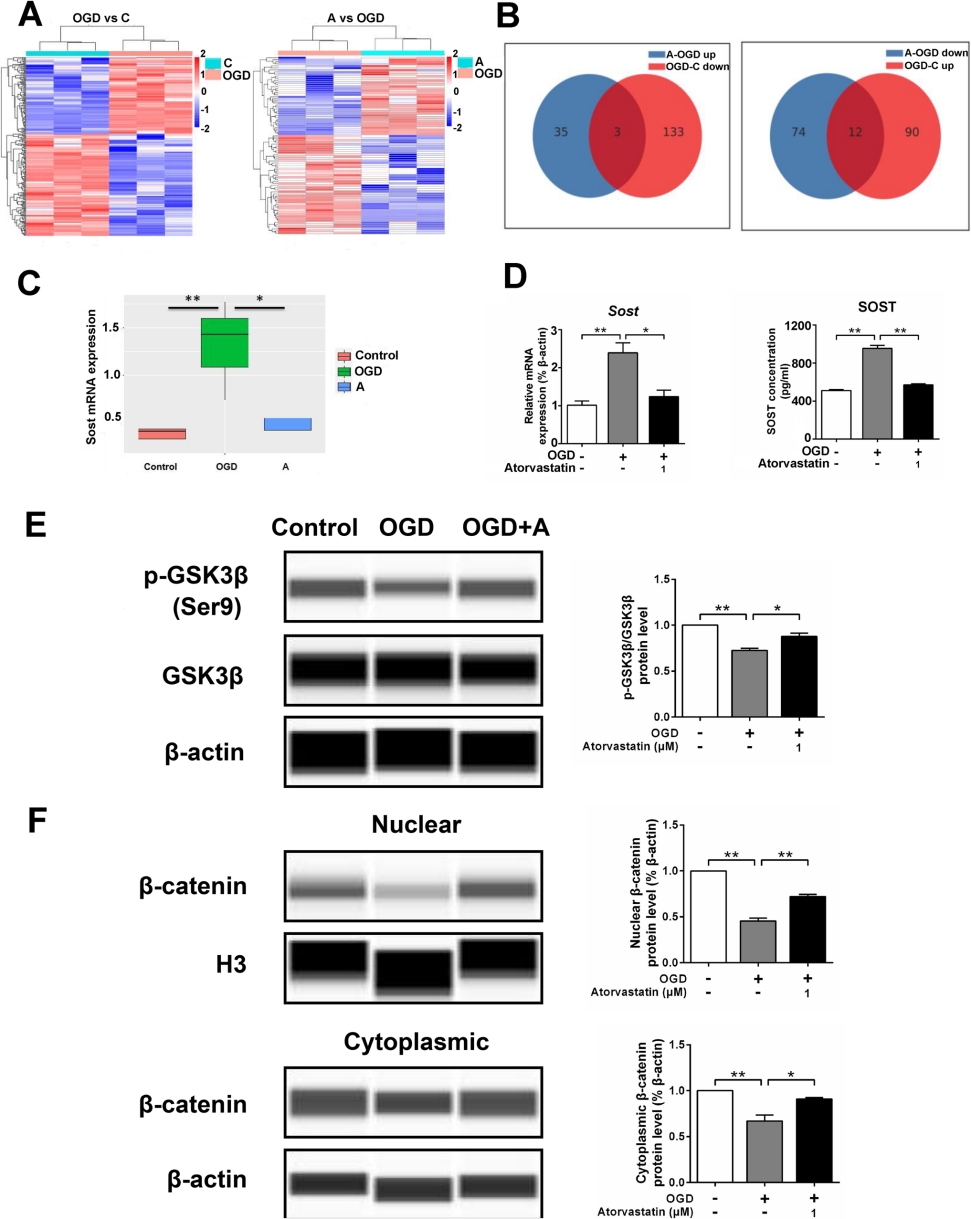
Sequencing analysis of microglia transcriptome. **A** Cluster map, relatively highly expressed protein-coding genes are shown in red and relatively low-expressed protein-coding genes are shown in blue. **B** The number of shared and unique differentially expressed genes between different comparison groups. **C** Transcriptome analysis of *Sost* mRNA expression. **D** The cell mRNA and cell culture supernatant protein level of SOST were detected by RT-qPCR and ELISA, respectively, *n* = 3/group. **E**, **F** The protein levels of p-GSK3β, GSK3β, and β-catenin were detected by the capillary western blot test, *n* = 3/group. Values represent the mean ± SEM. ^*^p < 0.05, ^**^p < 0.01 vs. the respective control

We sequenced the transcriptome of primarily cultured microglia and found that there were a number of significantly different genes between the OGD and control groups, as well as between the OGD and atorvastatin groups. There were three genes with decreased expression in the OGD group compared with the control group, while it was increased in the atorvastatin group, and 12 genes with increased expression in the OGD group compared with the control group but decreased in the atorvastatin group (Fig. 2A, B). Among the above differently expressed genes, the expression of genes decreased in the OGD group and increased in the atorvastatin group or increased in the OGD group and decreased in the atorvastatin group were mainly analyzed. We found that the expression of *Sost* mRNA increased significantly in the OGD group compared with the control group (fold change = 3.43), while compared with the OGD group, the expression of *Sost* mRNA in the atorvastatin group was significantly decreased (fold change = 0.38) (Fig. 2C). Further bioinformatic analysis and literature review showed that SOST can inhibit the Wnt/β-catenin signaling pathway by antagonizing the binding of Wnt to its receptor [42], and the Wnt/β-catenin signaling pathway has been found to play crucial roles in regulating microglial function in brain damage [43]; therefore, we next focused on examining the regulating roles of SOST and Wnt/β-catenin signaling pathways in microglia phenotypic transformation. RT-qPCR showed that the expression of *Sost* mRNA increased in the OGD group compared with the control group and decreased after administration of atorvastatin. ELISA detection of cell culture supernatant also showed that the protein level of SOST increased in the OGD group compared with the control group and decreased after administration of atorvastatin (Fig. 2D). We then further detected Wnt/β-catenin signaling molecules and found that the expression of p-GSK3β/GSK3β and nuclear and cytoplasmic β-catenin decreased in the OGD group compared with the control group but increased after administration of atorvastatin (Fig. 2E, F).

### SOST Recombinant Protein Attenuated the Effect of Atorvastatin on Cultured Microglia

To confirm the effect of SOST, we administered SOST recombinant protein to treat microglia. It showed that compared with the atorvastatin group, the expression of p-GSK3β/GSK3β and nuclear and cytoplasmic β-catenin in the SOST group was lower (Fig. 3A, B). Immunofluorescence staining showed that compared with the atorvastatin group, the pro-inflammatory microglia (CD16^+^/Iba1^+^) increased, and the anti-inflammatory microglia (CD206^+^/Iba1^+^) decreased in the SOST group (Fig. 3C, D). SOST increased the mRNA expression of pro-inflammatory factors (*Inos*, *Tnfα*, *and Il6*) and decreased the mRNA expression of anti-inflammatory factors (*Arg1*, *Tgfb*, *and Mrc1*) compared with the atorvastatin group (Fig. 3E, G). ELISA assay of cell culture supernatant showed that the protein levels of pro-inflammatory factors (iNOS and TNFα) increased, and the protein levels of anti-inflammatory factors (ARG1 and TGFβ) decreased in the SOST group compared with those in the atorvastatin group (Fig. 3F, H). These results showed that SOST attenuated the effect of atorvastatin on cultured microglia.

**Fig, 3:**
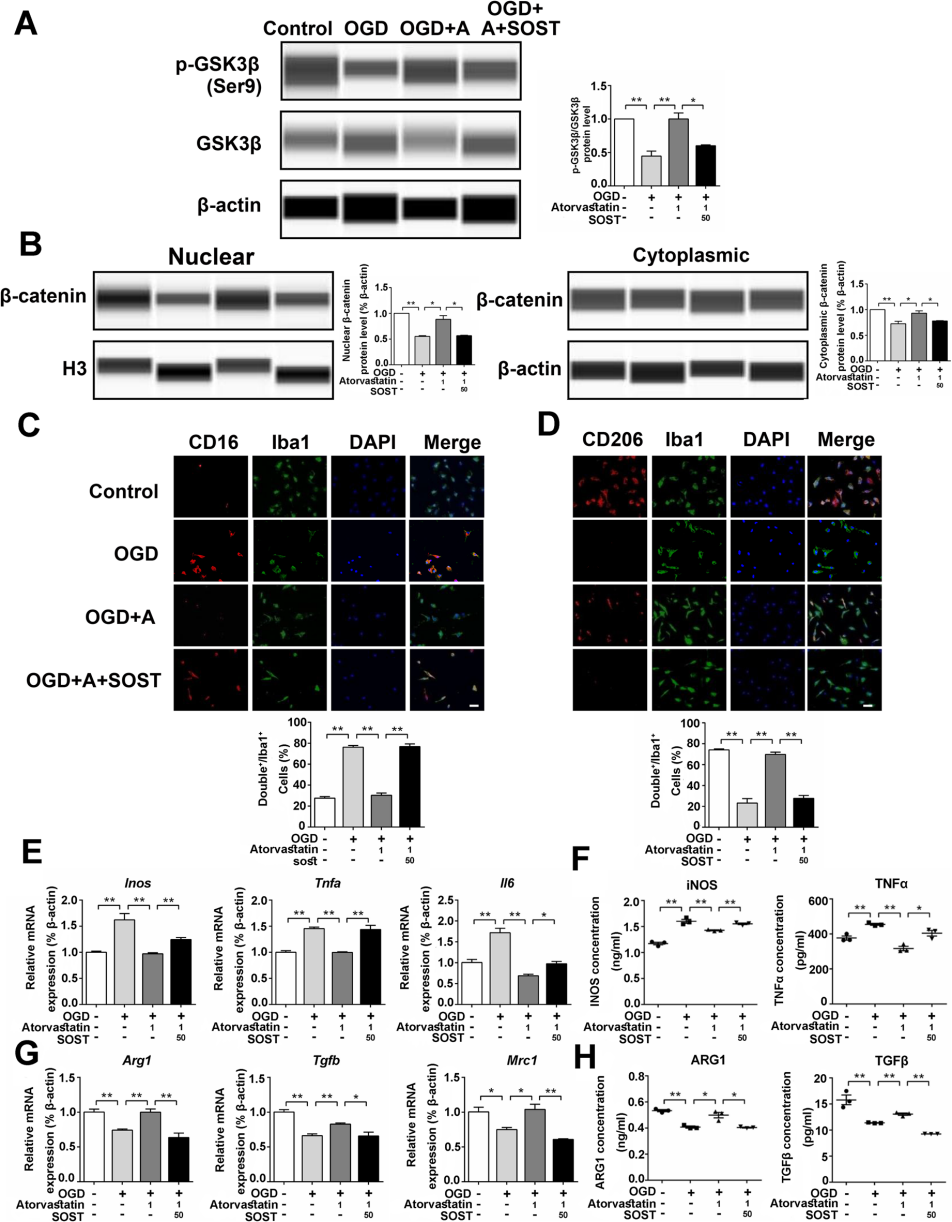
SOST recombinant protein attenuated the effect of atorvastatin on cultured microglia. **A**, **B** The protein levels of p-GSK3β, GSK3β, and β-catenin were detected by the capillary western blot test, *n* = 3/group. **C** Representative photographs of double immunofluorescence staining of CD16 (red) and Iba1 (green). Scale bar = 50 μm, *n* = 6/group. **D** Representative photographs of double immunofluorescence staining of CD206 (red) and Iba1 (green). Scale bar = 50 μm, *n* = 6/group. **E**, **G** The mRNA levels of *Inos*, *Tnfα*, *Il6*, *Arg1*, *Tgfb*, and *Mrc1* were detected by RT-qPCR, *n* = 3/group. **F**, **H** The cell culture supernatant protein levels of iNOS, TNFα, ARG1, and TGFβ were detected by ELISA, *n* = 3/group. Values represent the mean ± SEM. ^*^p < 0.05, ^**^p < 0.01 vs. the respective control

### XAV939 (Degradation of β-catenin) Attenuated the Effect of Atorvastatin on Cultured Microglia

**Fig. 4:**
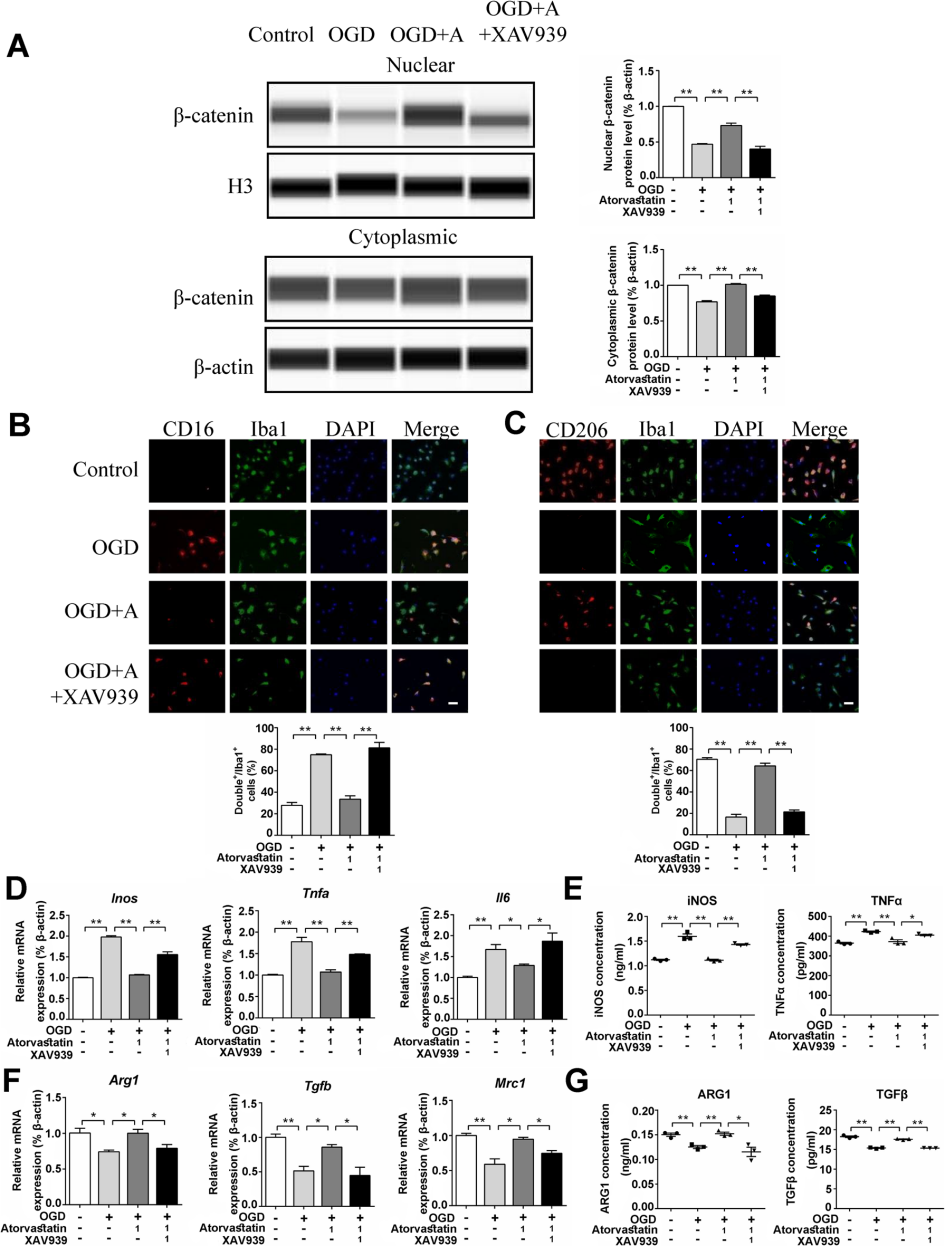
XAV939 (degradation of β-catenin) attenuated the effect of atorvastatin on cultured microglia. **A** The protein levels of β-catenin were detected by the capillary western blot test, n = 3/group. **B** Representative photographs of double immunofluorescence staining of CD16 (red) and Iba1 (green). Scale bar = 50 μm, *n* = 6/group. **C** Representative photographs of double immunofluorescence staining of CD206 (red) and Iba1 (green). Scale bar = 50 μm, *n* = 6/group. **D**, **F** The mRNA levels of *Inos*, *Tnfα*, *Il6*, *Arg1*, *Tgfb*, and *Mrc1* were detected by RT-qPCR, *n* = 3/group. **E**, **G** The cell culture supernatant protein levels of iNOS, TNFα, ARG1, and TGFβ were detected by ELISA, *n* = 3/group. Values represent the mean ± SEM. ^*^p < 0.05, ^**^p < 0.01 vs. the respective control

To confirm the role of the Wnt signaling pathway in regulating microglial function, we treated microglia with the β-catenin inhibitor XAV939 (degradation of β-catenin) and found that compared with the atorvastatin group, the β-catenin level in the nucleus and cytoplasm was reduced in the XAV939 group (Fig. 4A). Immunofluorescence staining showed that the levels of pro-inflammatory microglia (CD16^+^/Iba1^+^) increased, and the anti-inflammatory microglia (CD206^+^/Iba1^+^) decreased in the XAV939 group compared with the atorvastatin group (Fig. 4B, C). XAV939 also increased the mRNA levels of pro-inflammatory factors (*Inos*, *Tnfα*, *and Il6*) and reduced the mRNA levels of anti-inflammatory factors (*Arg1*, *Tgfb*, *and Mrc1*) compared with the atorvastatin group (Fig. 4D, F). ELISA of cell supernatants showed that the protein levels of pro-inflammatory factors (iNOS and TNFα) increased, and the protein levels of anti-inflammatory factors (ARG1 and TGFβ) decreased in the XAV939 group compared with the atorvastatin group (Fig. 4E, G). These results showed that the degradation of β-catenin attenuated the effect of atorvastatin on cultured microglia.

### Atorvastatin Alleviated HI-Induced Brain Injury and Neuroinflammation

**Fig. 5:**
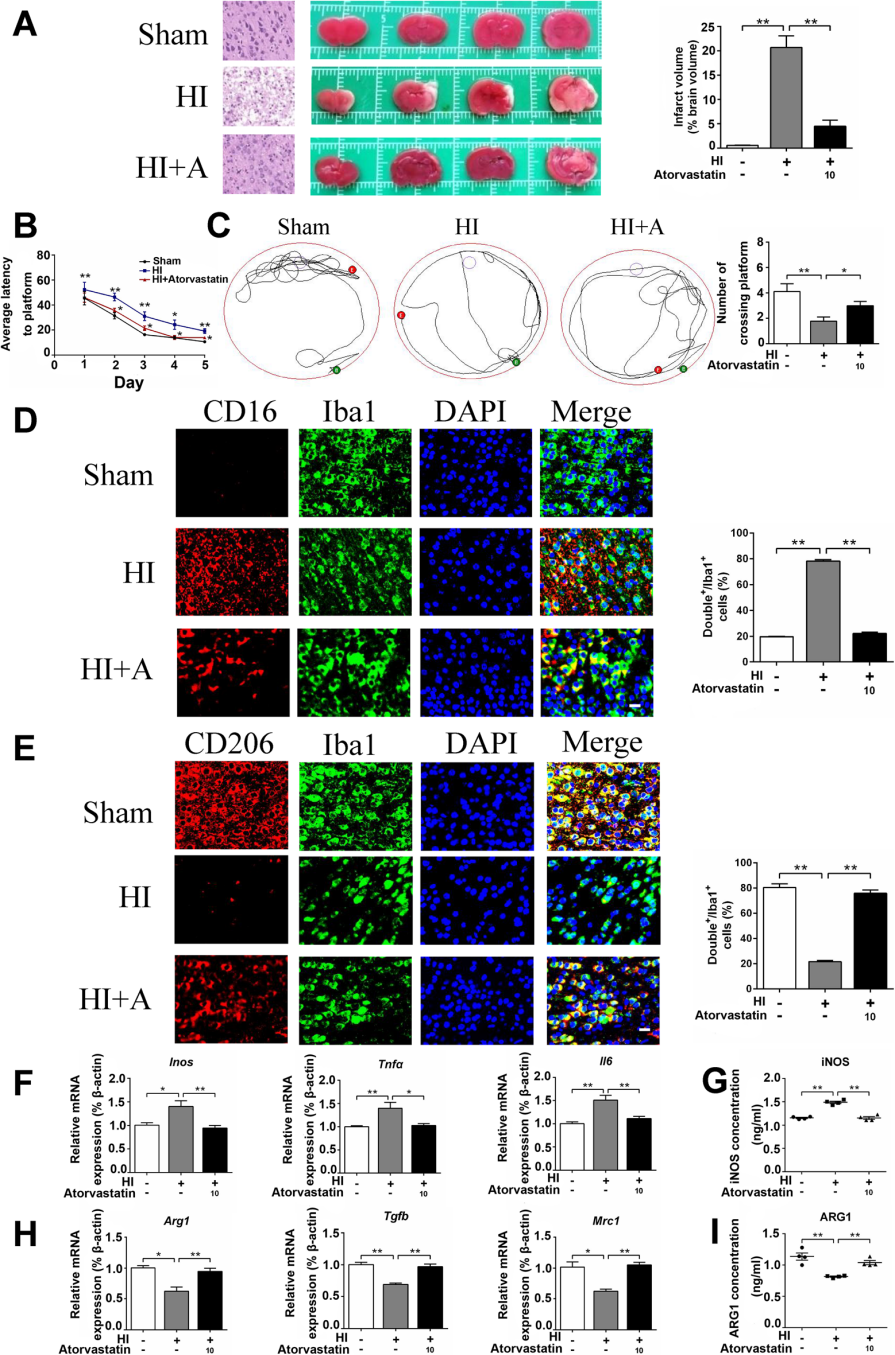
Atorvastatin alleviated HI-induced brain injury and neuroinflammation. **A** Pathological changes in the cerebral cortex (scale bar = 50 μm) and representative samples stained with TTC, *n* = 4/group. **B**, **C** Morris water maze test, *n* = 9/group. **D** Representative photographs of double immunofluorescence staining of CD16 (red) and Iba1 (green). Scale bar = 50 μm, *n* = 6/group. **E** Representative photographs of double immunofluorescence staining of CD206 (red) and Iba1 (green). Scale bar = 50 μm, *n* = 6/group. **F**, **H** The mRNA levels of *Inos*, *Tnfα*, *Il6*, *Arg1*, *Tgfb*, and *Mrc1* were detected by RT-qPCR, *n* = 6/group. **G**, **I** The protein levels of iNOS and ARG1were detected by ELISA, *n* = 4/group. Values represent the mean ± SEM. ^*^p < 0.05, ^**^p < 0.01 vs. the respective control

Atorvastatin was usually used in animal studies with the dosage of 10–20 mg/kg/d [33, 34]. In our previous study [44], we have carried out the concentration experiment with the dosage of 10 mg/kg/d and 20 mg/kg/d, respectively, and found that 20 mg/kg/d atorvastatin caused kidney and liver damage, while 10 mg/kg/d atorvastatin caused no significant kidney or liver injury. Therefore, the rats were treated with 10 mg/kg/d atorvastatin for present study. H&E staining showed that the cerebral cortex of the sham group was orderly arranged with normal cellular morphology. The HI group showed nuclear pyknosis and tissue dissolution in the cerebral cortex. Compared with the HI group, cortical tissue arrangement was more orderly, and nucleolus shrinkage was less in the atorvastatin treatment group. TTC staining showed that the volume of cerebral infarction increased in the HI group compared with the sham group, and atorvastatin treatment reduced the volume of cerebral infarction (Fig. 5A). The Morris water maze experiment in 28 day-old rats showed a longer average escape latency and fewer platform crossings in the HI group than in the sham group, while atorvastatin treatment decreased the average escape latency and increased the number of platform crossings (Fig. 5B, C).

We further examined the effect of atorvastatin on the regulation of microglial function in vivo. Immunofluorescence staining showed that compared with the sham group, the pro-inflammatory microglia (CD16^+^/Iba1^+^) increased, and the anti-inflammatory microglia (CD206^+^/Iba1^+^) decreased in the HI group. Compared with the HI group, atorvastatin treatment reduced pro-inflammatory microglia and increased anti-inflammatory microglia (Fig. 5D, E). We then detected inflammation-related indicators through RT-qPCR and found that compared with the sham group, the mRNA levels of pro-inflammatory factors (*Inos*, *Tnfα*, *and Il6*) increased in the HI group but decreased after atorvastatin treatment. The mRNA levels of anti-inflammatory factors (*Arg1*, *Tgfb*, *and Mrc1*) decreased in the HI group compared with the sham group but increased after atorvastatin treatment (Fig. 5F, H). Furthermore, ELISA showed that compared with the sham group, the protein level of pro-inflammatory factor (iNOS) increased in the HI group, while it was decreased after atorvastatin treatment. The protein level of anti-inflammatory factor (ARG1) decreased in the HI group compared with the sham group, while it was increased after atorvastatin treatment (Fig. 5G, I).

### Atorvastatin Affected Wnt/β-catenin Signaling Pathway in HIBD Rats

**Fig. 6:**
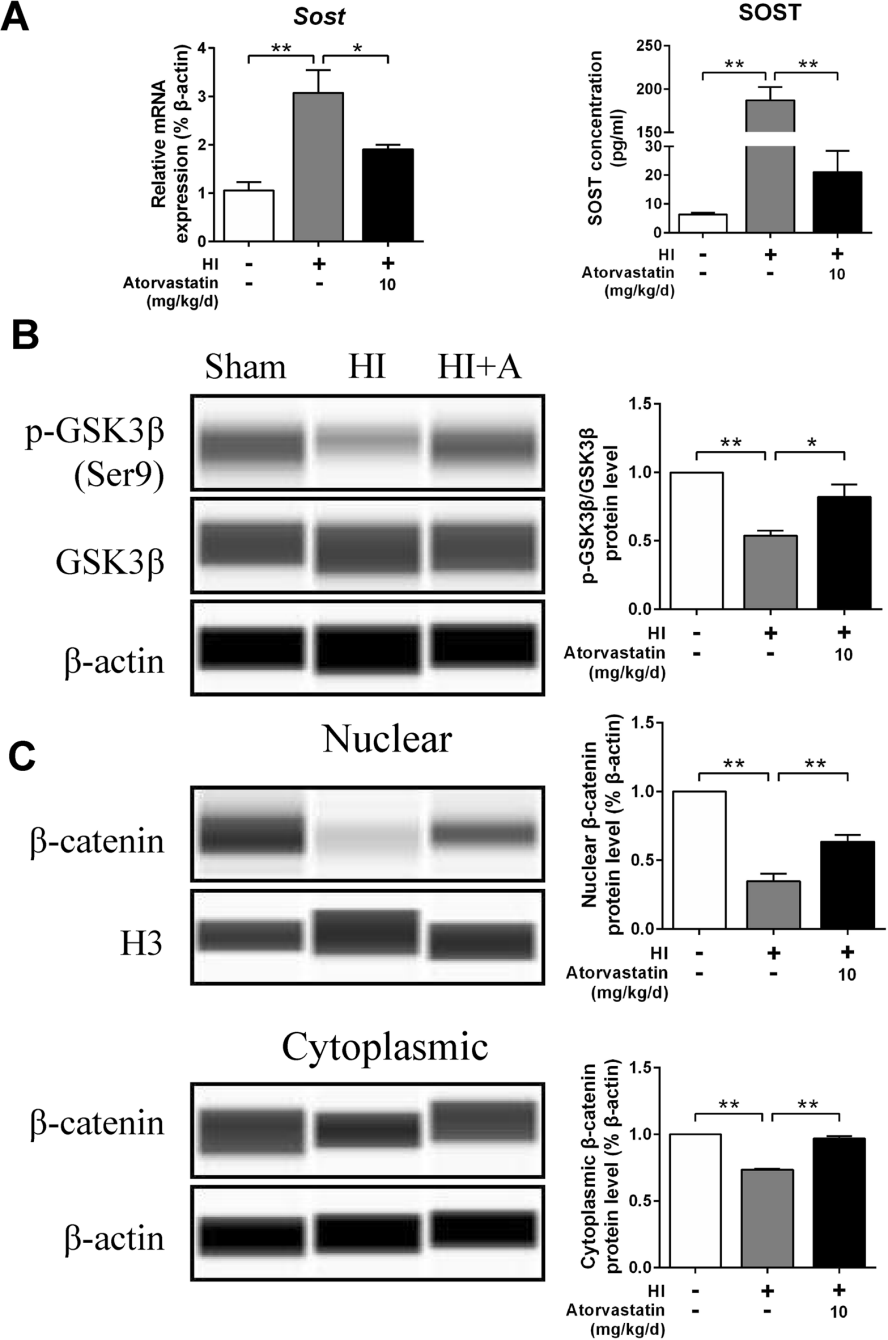
Atorvastatin affected Wnt/β-catenin signaling pathway in HIBD rats. **A** The protein level of SOST was detected by RT-qPCR (*n* = 6/group) and ELISA (*n* = 4/group), respectively. **B**, **C** The protein levels of p-GSK3β, GSK3β, and β-catenin were detected by the capillary western blot test, *n* = 4/group. Values represent the mean ± SEM. ^*^p < 0.05, ^**^p < 0.01 vs. the respective control

RT-qPCR and ELISA showed that HI increased the mRNA and protein expression of SOST, while atorvastatin treatment decreased the expression of SOST (Fig. 6A). Compared with the sham group, the expression of p-GSK3β/GSK3β and nuclear and cytoplasmic β-catenin protein expression decreased in the HI group but increased in the atorvastatin treatment group (Fig. 6B, C).

### SOST Recombinant Protein Attenuated the Protective Effect of Atorvastatin in HIBD Rats

**Fig. 7:**
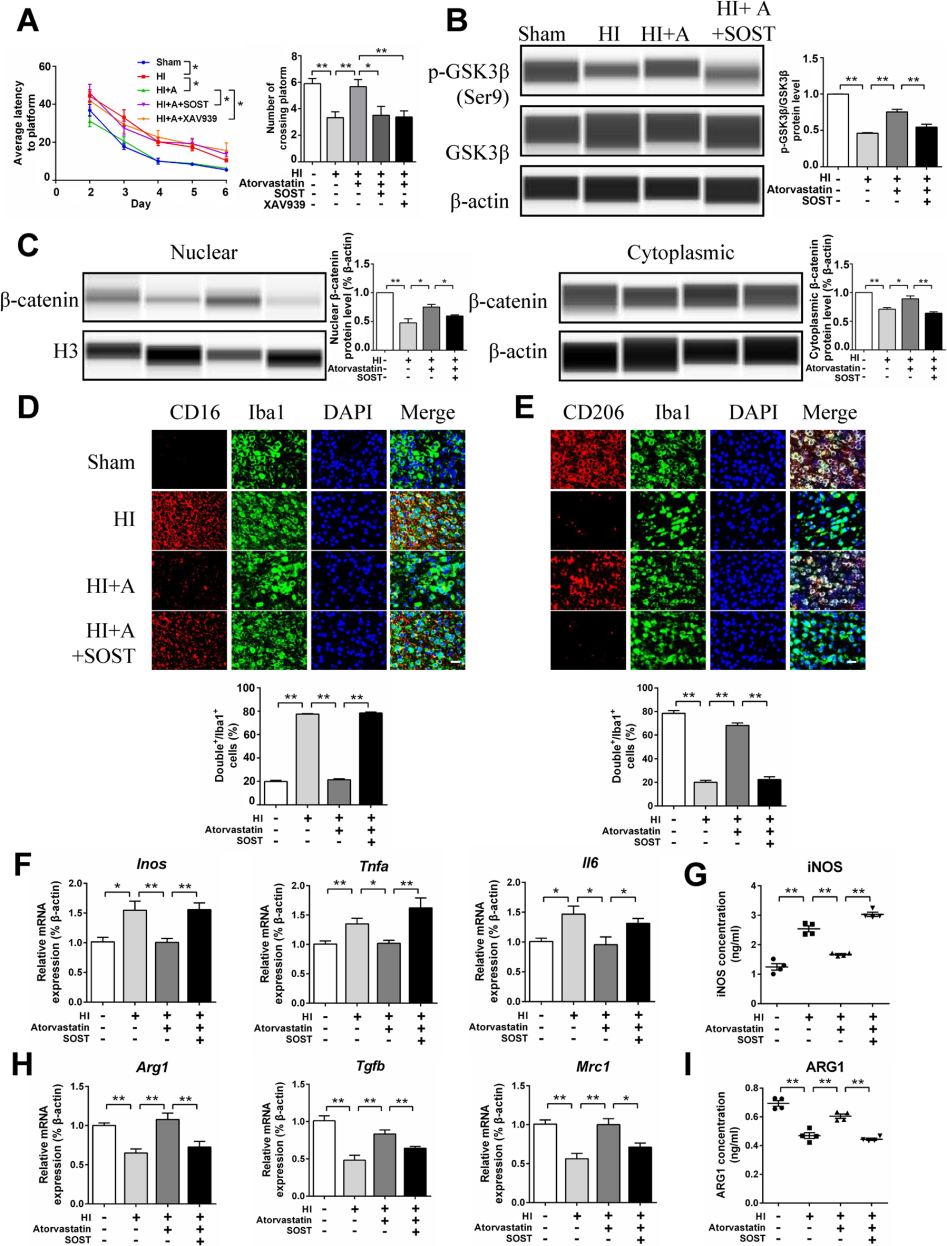
SOST recombinant protein attenuated the protective effect of atorvastatin in HIBD rats. **A** Morris water maze test, *n* = 8–9/group. **B**, **C** The protein levels of p-GSK3β, GSK3β, and β-catenin were detected by the capillary western blot test, *n* = 4/group. **D** Representative photographs of double immunofluorescence staining of CD16 (red) and Iba1 (green). Scale bar = 50 μm, *n* = 6/group. **E** Representative photographs of double immunofluorescence staining of CD206 (red) and Iba1 (green). Scale bar = 50 μm, *n* = 6/group. **F**, **H** The mRNA levels of *Inos*, *Tnfα*, *Il6*, *Arg1*, *Tgfb*, and *Mrc1* were detected by RT-qPCR, *n* = 6/group. **G**, **I** The protein levels of iNOS and ARG1were detected by ELISA, *n* = 4/group. Values represent the mean ± SEM. ^*^p < 0.05, ^**^p < 0.01 vs. the respective control

We further administered SOST recombinant protein (100 ng) through lateral ventricle injection in HIBD rats. Morris water maze experiment showed a longer average escape latency and lower number of platform crossings in the SOST group than in the atorvastatin group (Fig. 7A). The protein expression of p-GSK3β/GSK3β and nuclear and cytoplasmic β-catenin in the SOST group was lower than that in the atorvastatin group (Fig. 7B, C). Immunofluorescence staining showed that pro-inflammatory microglia (CD16^+^/Iba1^+^) increased and anti-inflammatory microglia (CD206^+^/Iba1^+^) decreased in the SOST group compared with the atorvastatin group (Fig. 7D, E). SOST increased the mRNA expression of pro-inflammatory factors (*Inos*, *Tnfα*, *and Il6*) and decreased the mRNA expression of anti-inflammatory factors (*Arg1*, *Tgfb*, *and Mrc1*) compared with the atorvastatin group (Fig. 7F, H). ELISA showed that the protein level of pro-inflammatory factor (iNOS) increased and the protein levels of anti-inflammatory factor (ARG1) decreased in the SOST group compared with the atorvastatin group (Fig. 7G, I). These results showed that SOST attenuated the protective effect of atorvastatin in HIBD rats.

### XAV939 (Degradation of β-catenin) Attenuated the Protective Effect of Atorvastatin in HIBD Rats

**Fig. 8:**
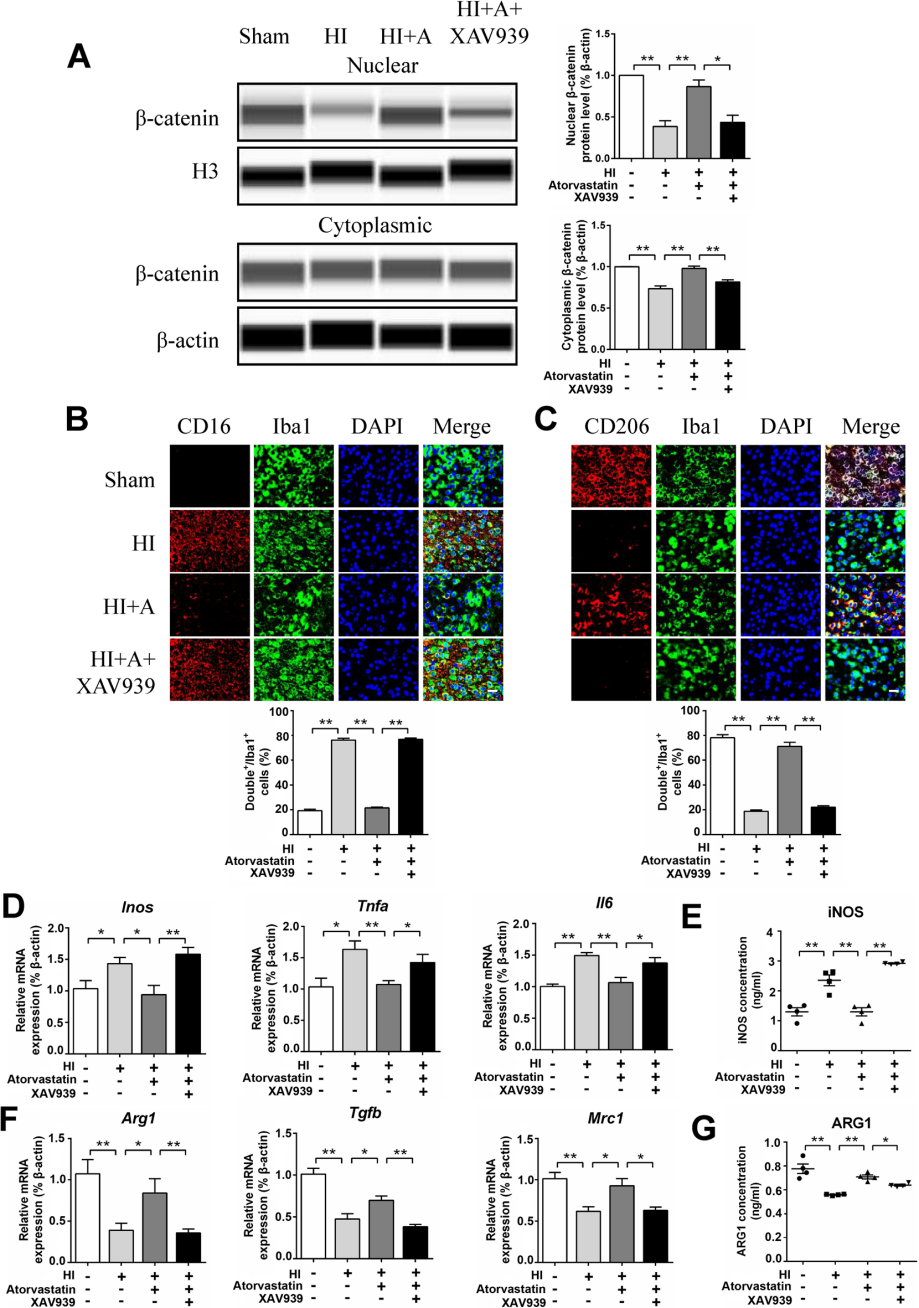
XAV939 (degradation of β-catenin) attenuated the protective effect of atorvastatin in HIBD rats. **A** The protein levels of β-catenin were detected by the capillary western blot test, *n* = 4/group. **B** Representative photographs of double immunofluorescence staining of CD16 (red) and Iba1 (green). Scale bar = 50 μm, *n* = 6/group. **C** Representative photographs of double immunofluorescence staining of CD206 (red) and Iba1 (green). Scale bar = 50 μm, *n* = 6/group. **D**, **F** The mRNA levels of *Inos*, *Tnfα*, *Il6*, *Arg1*, *Tgfb*, and *Mrc1* were detected by RT-qPCR, *n* = 6/group. **E**, **G** The protein levels of iNOS and ARG1were detected by ELISA, *n* = 4/group. Values represent the mean ± SEM. ^*^p < 0.05, ^**^p < 0.01 vs. the respective control

We further administered XAV939 (5 mg/kg) intraperitoneally in HIBD rats. Morris water maze experiment showed a longer average escape latency and lower number of platform crossings in the XAV939 group than in the atorvastatin group (Fig. 7A). The β-catenin level in the nucleus and cytoplasm was reduced in the XAV939 group (Fig. 8A). Immunofluorescence staining showed that pro-inflammatory microglia (CD16^+^/Iba1^+^) increased, while anti-inflammatory microglia (CD206^+^/Iba1^+^) decreased in the XAV939 group compared to the atorvastatin group (Fig. 8B, C). XAV939 also increased the mRNA levels of pro-inflammatory factors (*Inos*, *Tnfα*, *and Il6*) and reduced those of anti-inflammatory factors compared with the atorvastatin group (*Arg1*, *Tgfb*, *and Mrc1*) (Fig. 8D, F). ELISA revealed that the pro-inflammatory factor (iNOS) protein level increased, and the anti-inflammatory factor (ARG1) protein level decreased in the XAV939 group compared with the atorvastatin group (Fig. 8E, G). Therefore, the degradation of β-catenin was proven to attenuate the protective effect of atorvastatin in HIBD rats.

## Discussion

This study revealed that administration of atorvastatin in HIBD rats reduced cerebral infarction volume, improved learning and memory ability of rats, decreased pro-inflammatory microglia and pro-inflammatory factors, and increased anti-inflammatory microglia and anti-inflammatory factors. To the best of our knowledge, this study is the first to demonstrate that atorvastatin reduces pro-inflammatory microglia and increases anti-inflammatory microglia in a rat HIBD model. We further demonstrated that HI inhibited the Wnt/β-catenin signaling pathway by enhancing SOST expression, thereby increasing pro-inflammatory microglia and pro-inflammatory factors. Administration of atorvastatin inhibited the expression of SOST, thereby activating the Wnt/β-catenin signaling pathway and promoting pro-inflammatory to anti-inflammatory transformation of microglia, ultimately improving the microenvironment of neuroinflammation and attenuated brain injury.

Microglia can be transformed between two phenotypes (pro-inflammatory and anti-inflammatory); pro-inflammatory microglia express CD16 and CD86 and release inflammatory cytokines, such as IL6 and TNFα, thus inducing brain damage [15, 16]. Anti-inflammatory microglia express CD206 and ARG1 and release anti-inflammatory cytokines and growth factors, such as TGFβ, thereby exerting a neuroprotective effect [17, 18]. Studies have shown that the transformation of microglia from the pro-inflammatory phenotype to the anti-inflammatory phenotype attenuated neuroinflamation and promoted the recovery of a series of brain disease such as stroke, Alzheimer’s disease, and Parkinson’s disease [45–47].

Neuroinflammation has also been found to play a key role in HI-induced brain damage. HI activates microglia, and activated microglia promote neuroinflammation and cause brain damage [22]. In the adult mouse middle cerebral artery occlusion model, microglia and macrophages are activated, cytokines are produced in large quantities, and the level of inflammatory factors is increased [48, 49]. In a neonatal rat model of hypoxia-ischemia, microglia are activated, and inflammatory factors are increased [50]. In a neonatal rat model of middle cerebral artery occlusion, macrophages and microglia are activated, and pro-inflammatory cytokines are increased [51]. In this study, we found that HI significantly increased pro-inflammatory microglia and the secretion of pro-inflammatory factors and reduced anti-inflammatory microglia and the secretion of anti-inflammatory factors, thereby causing brain damage.

Atorvastatin can reduce cholesterol synthesis by inhibiting HMG-CoA and is a commonly used lipid-lowering drug in clinical practice [52]. Studies have shown that oral gavage statins can pass through the blood-brain barrier (BBB) to reach the cerebral and play a neuroprotective role [53]. In an epilepsy rat model, after 7 days of treatment with atorvastatin (10 mg/kg/d, po), the plasma and cerebral cortex levels of atorvastatin reached 19.97 ± 6.33 pmol/ml and 179.92 ± 15.31 pmol/g, respectively [54]. In addition, cerebral ischemia can increase BBB permeability, which is conducive to the passage of atorvastatin [55]. In this study, we found that intragastrically administered atorvastatin can exert a neuroprotective effect by reducing inflammation in HIBD rats, suggesting that intragastrically administered atorvastatin can reach the brain through the BBB in the HIBD model.

The Wnt/β-catenin pathway includes several members of the Frizzled protein family and LRP5/6, whose activation leads to the inactivation of glycogen synthase kinase3β (GSK3β) [56]. Ser9 phosphorylation of GSK3β is the inactive state of GSK3β [57]. The increase in inactivated GSK3β reduces the degradation of β-catenin in the cytoplasm, resulting in the increase of β-catenin in the cytoplasm, and the increased β-catenin enters the nucleus and activates the Wnt signaling pathway [57, 58]. The Wnt/β-catenin pathway is a key regulator involved in maintaining neuronal homeostasis [59]. It has also been found to play an important role in the regulation of microglial function in brain injury [43]. Many studies have shown that activation of the Wnt/β-catenin signaling pathway can inhibit pro-inflammatory microglia and pro-inflammatory factors and increase anti-inflammatory microglia and anti-inflammatory factors in brain injuries such as subarachnoid hemorrhage, glioblastoma, and depression [39, 43, 60].

Previous studies have revealed that atorvastatin can activate Wnt/β-catenin signaling pathway in rat osteoporotic periodontitis model and human breast cancer cell line [37, 38]. Furthermore, SOST can antagonize the binding of Wnt to its receptor, reducing the expression of β-catenin and thus inhibiting the Wnt signaling pathway [41]. When the Wnt signaling pathway is inhibited, GSK3β is activated. GSK-3β can degrade IkB (a major inhibitor of NF-κB), resulting in reduced NF-κB degradation and increased nuclear translocation and finally induces pro-inflammatory gene cascade that increases the transition of microglia to pro-inflammatory phenotype [61–63]. In this study, we found that HI induced the expression of SOST and thus inhibited the Wnt/β-catenin signaling pathway, thereby increasing pro-inflammatory microglia and secretion of pro-inflammatory factors. However, atorvastatin reduced the expression of SOST, thus activating the Wnt/β-catenin signaling pathway, increasing anti-inflammatory microglia, secreting anti-inflammatory cytokines, and alleviated brain damage.

Based on the above findings, a promising therapeutic goal of improving brain damage outcomes can be achieved by focusing on the pro/anti-inflammatory conversion process of HI. Furthermore, Wnt/β-catenin signaling may be a promising target for HIE treatment in neonates and is applicable to other diseases involving neuroinflammation.

This study has some limitations. In this study, we focused on the effect of atorvastatin on pro/anti-inflammatory phenotypic transformation of microglia after HI. However, there are other types of cells in the brain; among them, astrocytes are also inflammation-related cells. Tian et al. [64] have found that in the mouse sepsis model, atorvastatin reduced the proliferation of astrocytes in the hippocampus and thus reduced brain damage. Pannu et al. [65] and Bimbova et al. [66] have found that in adult rat models of spinal cord injury, atorvastatin attenuated spinal cord injury through regulating astrocyte function. These studies were conducted in adult animal models, and the effect of atorvastatin on astrocytes in developing brain injury is not clear. The effect of atorvastatin on astrocyte function during HIBD needs to be further studied. Besides, we can not exclude that systemic effect of atorvastatin on cholesterol synthesis may be influencing the atorvastatin-induced effects. One previous study has revealed that higher cholesterol is associated with impaired white matter development and worse motor outcomes in preterm newborns [67]. Puglielli et al. [68] have found that increased cholesterol can lead to the development of Alzheimer’s disease. These researches suggest that atorvastatin might affect brain function through modulating cholesterol metabolism, which is worth exploring in the future.

## Conclusions

In summary, the present study revealed that atorvastatin promoted pro-inflammatory to anti-inflammatory transformation of microglia via the Wnt/β-catenin pathway in hypoxic-ischemic neonatal rats, rendering it an optimal choice to treat neonatal HIE. Atorvastatin is a widely used statin lipid-lowering drug in clinical practice, and its safety has been confirmed in clinic practice. Taken together, we believe that atorvastatin might be developed as a potent agent to treat neonatal HIE in the future.

## Data Availability

The datasets used and/or analyzed during the current study are available from the corresponding authors on reasonable request.

## Abbreviations

HIE: Hypoxic-ischemic encephalopathy
HIBD: Hypoxic-ischemic brain damage
H&E: Hematoxylin and eosin
IL: Interleukin
CNS: Central nervous system
TNFα: Tumor necrosis factor α
iNOS: Inducible nitric oxide synthase
TGFβ: Transforming growth factor β
OGD: Oxygen glucose deprivation
TTC: Triphenyltetrazolium chloride monohydrate
SOST: Sclerostin
BBB: Blood-brain barrier
Arg1: Arginase 1
Mrc1: Mannose receptor C-type 1
HMG-CoA: 3-Hydroxy-3-methylglutaryl coenzyme A
SD: Sprague Dawley
